# Systemic Anti-Inflammatory and Immunomodulatory Effects of Intravenous Lidocaine During Robotic-Assisted Radical Prostatectomy: A Prospective Observational Study

**DOI:** 10.3390/medicina62010068

**Published:** 2025-12-28

**Authors:** Georgiana Maria Popa, Simona Alina Abu-Awwad, Ahmed Abu-Awwad, Carmen-Ioana Marta, Erika Bimbo-Szuhai, Mihaela Gabriela Bontea, Adrian Gheorghe Osiceanu, Cristian Mihai Moisa Cezar, Ciprian Dumitru Puscas, Teodor Traian Maghiar, Iulia Codruta Macovei, Mihai O. Botea

**Affiliations:** 1 Pelican Hospital, Corneliu Coposu Street 2, 410450 Oradea, Romania; pascalau.georgianamaria@student.uoradea.ro (G.M.P.); cipripuski@yahoo.com (C.D.P.); mbotea@uoradea.ro (M.O.B.); 2 Department of Surgical Disciplines, Faculty of Medicine and Pharmacy, University of Oradea, 1st December Square 10, 410073 Oradea, Romania; cmacovei@uoradea.ro; 3 Doctoral School of Biomedical Sciences, University of Oradea, 410087 Oradea, Romania; moisa.cezarcristianmihai@student.uoradea.ro; 4 Department of Obstetrics and Gynecology, “Victor Babes” University of Medicine and Pharmacy Timisoara, Eftimie Murgu Square 2, 300041 Timisoara, Romania; alina.abuawwad@umft.ro (S.A.A.-A.); carmen.marta@umft.ro (C.-I.M.); 5 Clinic of Obstetrics and Gynecology, “Pius Brinzeu” County Clinical Emergency Hospital, 300723 Timisoara, Romania; 6 Department XV—Discipline of Orthopedics—Traumatology, “Victor Babes” University of Medicine and Pharmacy, Eftimie Murgu Square, No. 2, 300041 Timisoara, Romania; ahm.abuawwad@umft.ro; 7 Research Center, University Professor Doctor Teodor Șora, “Victor Babes” University of Medicine and Pharmacy, Eftimie Murgu Square, No. 2, 300041 Timisoara, Romania; 8 Department of Morphological Disciplines, Faculty of Medicine and Pharmacy, University of Oradea, 1st December Square 10, 410073 Oradea, Romania; bontea.mihaela@yahoo.ro (M.G.B.); osiceanuadrian@yahoo.com (A.G.O.); uro_doruletul@yahoo.com (T.T.M.)

**Keywords:** intravenous lidocaine, robotic-assisted radical prostatectomy, perioperative inflammation, cytokine dynamics, IL-6, CRP, fibrinogen, immunomodulation, postoperative pain, ERAS protocol

## Abstract

*Background and Objectives*: Surgical stress during robotic-assisted radical prostatectomy (RARP) elicits a measurable systemic inflammatory response despite the minimally invasive approach. Intravenous lidocaine has been increasingly investigated for its potential anti-inflammatory, analgesic, and immunomodulatory benefits, but evidence in robotic urologic oncology remains limited. This study aimed to evaluate whether intraoperative lidocaine infusion attenuates postoperative inflammation, improves analgesic outcomes, and accelerates early recovery following RARP. *Materials and Methods*: This prospective non-randomized observational study included 80 patients undergoing elective RARP, divided into a Lidocaine Group (*n* = 40) receiving an intraoperative bolus and continuous infusion, and a Control Group (*n* = 40) receiving standard anesthesia without lidocaine. Serum IL-6, TNF-α, CRP, and fibrinogen were measured at baseline, end of surgery, and 24 h postoperatively. Postoperative pain scores, opioid consumption, gastrointestinal recovery, ambulation, and length of stay were recorded. Statistical analyses included repeated-measures ANOVA, correlation testing, and between-group comparisons. *Results*: Baseline characteristics were similar between groups. At 24 h postoperatively, lidocaine administration was associated with a significantly attenuated inflammatory response, with lower levels of IL-6 (45.7 ± 10.8 vs. 68.9 ± 12.6 pg/mL, *p* < 0.01) and TNF-α (20.5 ± 5.1 vs. 27.2 ± 6.4 pg/mL, *p* < 0.01) compared with controls. Patients receiving lidocaine reported lower postoperative pain scores and required significantly less opioid analgesia, with a total 24 h consumption of 8.9 ± 3.4 vs. 14.8 ± 5.2 mg morphine milligram equivalents (*p* < 0.001). Lidocaine was also associated with faster recovery, including earlier oral intake and a shorter length of hospital stay (2.9 ± 0.7 vs. 3.6 ± 0.9 days, *p* = 0.003). No lidocaine-related adverse events were observed. *Conclusions*: In this prospective observational study, intraoperative intravenous lidocaine was associated with attenuated early postoperative inflammation, improved analgesic outcomes, and enhanced early recovery following RARP. These findings support the potential role of intravenous lidocaine as a safe adjunct in multimodal perioperative management; however, given the non-randomized observational design, causal inferences should be interpreted with caution. Further randomized controlled trials are warranted to confirm causality and to validate long-term clinical and mechanistic effects. Potential residual confounding inherent to the observational design should be considered when interpreting these findings.

## 1. Introduction

Surgical stress and the ensuing inflammatory cascade represent pivotal challenges in perioperative medicine, particularly in oncologic surgery [1]. Even in the era of minimally invasive and robotic-assisted procedures, the biological response to surgery extends far beyond the visible tissue trauma. It involves a complex interplay between neural, endocrine, and immune pathways that, if left unchecked, can contribute to postoperative pain, delayed recovery, and in some cases, adverse oncologic outcomes [2]. Managing this systemic response has therefore become a cornerstone of modern anesthetic and perioperative care.

Among the pharmacological strategies proposed to modulate surgical stress, intravenous lidocaine has emerged as one of the most intriguing and versatile agents [3]. Traditionally recognized as a local anesthetic and antiarrhythmic drug, lidocaine has demonstrated systemic anti-inflammatory and immunomodulatory properties when administered intravenously at subanesthetic doses [4]. Its actions extend well beyond sodium channel blockade, involving modulation of NMDA receptors, suppression of cytokine release, and attenuation of neutrophil activation. Through these mechanisms, lidocaine may reduce not only nociceptive transmission but also the inflammatory and oxidative stress associated with surgical trauma [5].

Robotic-assisted radical prostatectomy represents a paradigm of advanced, minimally invasive oncologic surgery. Despite the smaller incisions and lower tissue trauma compared with open procedures, the physiological stress associated with pneumoperitoneum, prolonged Trendelenburg positioning, and neurovascular manipulation can still trigger systemic inflammation and transient immune dysregulation [6]. Elevated levels of interleukin-6 (IL-6), tumor necrosis factor-alpha (TNF-α), and C-reactive protein (CRP) have been documented even after robotic procedures, reflecting that surgical “minimally invasiveness” does not equate to immunological neutrality. Elevated fibrinogen levels have also been described as part of the acute-phase reaction following major surgery, reflecting the intensity of the systemic inflammatory response and its link with coagulation dynamics. This transient proinflammatory state can influence postoperative recovery, pain perception, and potentially the biological milieu in which residual cancer cells may survive or recur [7].

In this context, lidocaine has attracted increasing interest not only as an analgesic adjunct but also as a perioperative immunomodulator. Several experimental and clinical studies have suggested that lidocaine can blunt the postoperative rise in IL-6, inhibit neutrophil extracellular trap formation, and preserve lymphocyte function. These effects are thought to promote a more balanced immune response, mitigating the exaggerated inflammatory surge without impairing essential defense mechanisms. Such modulation may translate into tangible clinical benefits: less pain, lower opioid requirements, faster recovery, and a potentially more favorable postoperative immunologic profile [8,9,10,11,12].

However, despite compelling evidence from abdominal and gynecologic surgery, data on lidocaine’s systemic anti-inflammatory effects in robotic-assisted urologic surgery remain scarce. The unique physiological environment of robotic prostatectomy, characterized by elevated intra-abdominal pressure, limited visceral manipulation, and distinct hemodynamic changes, warrants specific investigation. Understanding how lidocaine influences the perioperative inflammatory trajectory in this setting could refine multimodal strategies that combine effective analgesia with enhanced biological recovery.

This study was designed to further explore the immunomodulatory potential of intravenous lidocaine in the setting of robotic-assisted prostatectomy. Building on prior evidence of its analgesic and opioid-sparing properties, the present research focuses on how lidocaine influences systemic inflammation and cytokine dynamics during the perioperative period. By monitoring changes in IL-6, CRP, TNF-α, and fibrinogen, and by correlating these biomarkers with clinical indicators of recovery, we sought to capture both the biological and functional impact of this intervention.

We hypothesized that intraoperative lidocaine infusion attenuates the systemic inflammatory response, reduces postoperative pain and opioid consumption, and promotes a more balanced physiological recovery following robotic-assisted surgery.

Such findings could reinforce the integration of lidocaine into enhanced recovery pathways for robotic urologic oncology, aligning symptom control with the preservation of immune and biological homeostasis in surgical care.

## 2. Materials and Methods

### 2.1. Study Design and Ethical Approval

This prospective observational study was carried out at Pelican Clinical Hospital in Oradea, Romania, a tertiary center with expertise in minimally invasive and robotic urologic procedures. Adult male patients scheduled to undergo robotic-assisted radical prostatectomy (RARP) for biopsy-confirmed prostate adenocarcinoma were consecutively recruited between November 2021 and June 2025.

The study protocol received approval from the Pelican Clinical Hospital Ethics Committee (Approval No. 2166/19 October 2021). All research activities complied with the ethical principles outlined in the Declaration of Helsinki (2013 edition) and adhered to European Good Clinical Practice (GCP) standards. Before enrollment, each participant provided written informed consent after being informed about the study objectives and procedures. Given the observational and non-randomized design, the study was not intended to establish causality but rather to explore associations between intraoperative lidocaine administration, inflammatory biomarkers, and early postoperative recovery.

Group allocation was based on a predefined institutional analgesic protocol implemented during the study period. Patients were assigned to the Lidocaine or Control group according to the standard perioperative protocol in use at the time of surgery, and not based on individual patient characteristics or intraoperative clinical judgment.

Confounding was addressed through strict inclusion and exclusion criteria, standardized anesthetic and surgical management, and detailed baseline characterization with between-group comparisons of demographic, clinical, and perioperative variables. Given the sample size and the exploratory nature of the study, no multivariable adjustment was performed.

### 2.2. Study Population

**Figure 1:**
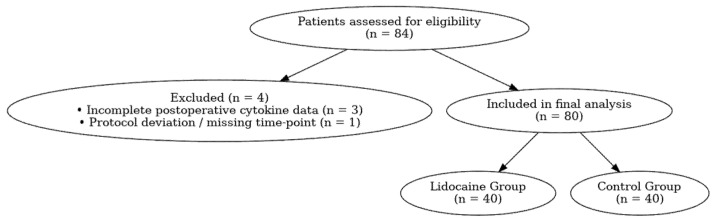
Study flow diagram and patient selection process.

A total of 84 consecutive patients scheduled for elective RARP were screened for eligibility, and 80 patients were included in the final analysis (Figure 1). Eligible participants were men aged 40–80 years, diagnosed with localized prostate adenocarcinoma (clinical stages T1–T2, N0, M0) and classified as ASA physical status I–III according to the American Society of Anesthesiologists.

Exclusion criteria comprised known allergy or contraindication to lidocaine, chronic opioid use or neuropathic pain disorders, severe hepatic, renal, or cardiac dysfunction (ejection fraction < 40%, eGFR < 45 mL/min/1.73 m^2^, or Child–Pugh class B/C), conversion to open surgery, intraoperative complications, or incomplete follow-up or laboratory data.

Patients were divided into two comparable groups based on the intraoperative analgesic protocol: the Lidocaine Group (LG, *n* = 40), which received an intravenous lidocaine bolus followed by continuous infusion, and the Control Group (CG, *n* = 44), which underwent identical anesthetic management without lidocaine administration.

**Figure 2:**
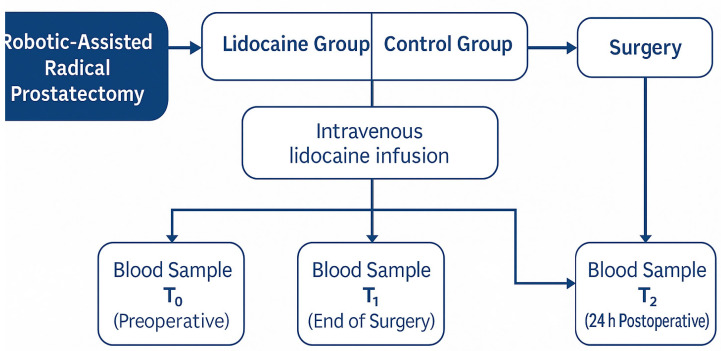
Study design and perioperative sampling timeline in patients undergoing robotic-assisted radical prostatectomy.

The overall study workflow and sampling schedule are illustrated in Figure 2. This schematic summarizes patient allocation into the two study groups, the timing of intravenous lidocaine administration, and the perioperative blood sampling points (T_0_, T_1_, and T_2_) for biomarker analysis.

### 2.3. Anesthetic and Perioperative Management

All procedures were performed using the da Vinci Xi robotic system by the same senior surgical team at Pelican Clinical Hospital, Oradea, ensuring consistency in operative technique. To reduce inter-patient variability, a standardized general anesthesia regimen was implemented for every case. Induction was achieved with intravenous propofol, fentanyl, and rocuronium, and anesthesia was maintained with sevoflurane in a mixture of oxygen and air. Additional doses of fentanyl were administered intraoperatively when required to keep hemodynamic values within approximately ±20% of each patient’s baseline measurements.

Throughout the intervention, patients were monitored continuously by electrocardiography, pulse oximetry, capnography, and noninvasive arterial blood pressure assessment. Mechanical ventilation was provided in volume-controlled mode, with tidal volumes set between 6 and 8 mL/kg and a positive end-expiratory pressure (PEEP) of 5 cmH_2_O [13].

Intraoperative analgesia consisted exclusively of fentanyl, administered at induction and supplemented as required to maintain hemodynamic stability within ±20% of baseline values. Total intraoperative fentanyl consumption was recorded for all patients. No additional opioid agents or adjunct analgesic or anti-inflammatory therapies, including ketamine, dexmedetomidine, gabapentinoids, acetaminophen, NSAIDs, regional anesthesia techniques (e.g., TAP blocks), or local anesthetic infiltration, were routinely used as part of the standardized anesthetic protocol.

### 2.4. Lidocaine Infusion Protocol

Patients allocated to the Lidocaine Group were administered an intravenous loading dose of lidocaine at 1.5 mg/kg immediately after anesthetic induction. This was followed by a continuous infusion delivered at 1.5 mg/kg/h for the duration of the surgical procedure. The cumulative amount of lidocaine administered to any participant was capped at a maximum of 300 mg.

No postoperative lidocaine infusion was continued. The Control Group received an equivalent volume of saline solution administered with identical timing and infusion rates to ensure procedural consistency during intraoperative management, rather than as a formal placebo intervention [14].

### 2.5. Blood Sampling and Cytokine Measurement

Peripheral venous blood samples were collected at three predefined time points:

- T_0_ (baseline): immediately before anesthesia induction.
- T_1_ (end of surgery): within 10 min after skin closure.
- T_2_ (24 h postoperatively).

Serum was separated and stored at −80 °C until analysis.

Levels of IL-6, TNF-α, and CRP were measured using enzyme-linked immunosorbent assay (ELISA) kits (R&D Systems, Minneapolis, MN, USA) according to manufacturer instructions. All assays were performed in duplicate by technicians blinded to patient allocation. Fibrinogen concentrations were determined using an immunoturbidimetric assay (Roche Diagnostics, Basel, Switzerland) on the same samples collected at T_0_, T_1_, and T_2_.

Serum concentrations of IL-6, TNF-α, and CRP were determined using commercially available enzyme-linked immunosorbent assay (ELISA) kits (R&D Systems, Minneapolis, MN, USA). Specifically, IL-6 was measured using the Human IL-6 Quantikine ELISA Kit (Catalog No. D6050; analytical sensitivity < 0.70 pg/mL), and TNF-α using the Human TNF-α Quantikine ELISA Kit (Catalog No. DTA00D; analytical sensitivity < 1.6 pg/mL).

All assays were performed according to the manufacturer’s instructions, and samples were analyzed in duplicate by laboratory personnel blinded to group allocation. The intra-assay and inter-assay coefficients of variation were within the ranges reported by the manufacturer.

Fibrinogen concentrations were measured using an immunoturbidimetric assay on a Roche Diagnostics platform (Basel, Switzerland), following standard laboratory protocols.

### 2.6. Clinical Parameters and Postoperative Assessment

In addition to cytokine profiles, perioperative data included duration of surgery, anesthetic time, estimated blood loss, and length of hospital stay.

Postoperative pain intensity was evaluated using a 10-point Visual Analogue Scale (VAS) at 4, 12, and 24 h. Opioid consumption was recorded and converted to morphine milligram equivalents (MME). Early recovery parameters, including time to oral intake, gastrointestinal function recovery, and first ambulation, were also documented.

Safety monitoring and pharmacovigilance.

All patients receiving intravenous lidocaine were monitored continuously using standard intraoperative electrocardiography, pulse oximetry, and noninvasive blood pressure measurement, in accordance with institutional anesthesia protocols. Postoperatively, patients were clinically assessed for potential signs of local anesthetic systemic toxicity (LAST), including neurological symptoms (e.g., dizziness, perioral numbness, tinnitus, confusion) and cardiovascular manifestations (e.g., arrhythmias or hemodynamic instability). Routine serum lidocaine concentration monitoring was not performed, as the administered dosing regimen (1.5 mg/kg bolus followed by 1.5 mg/kg/h infusion, with a maximum cumulative dose of 300 mg) remained within widely accepted safety margins. No clinical signs of lidocaine-related toxicity or adverse events were observed throughout the perioperative period.

### 2.7. Data Collection and Quality Control

Clinical, laboratory, and anesthetic data were extracted from electronic hospital records by two independent investigators blinded to group allocation. Cytokine measurements were performed by the same laboratory personnel to reduce inter-assay variability. Data consistency was verified by cross-checking between digital and paper records, and discrepancies were resolved by consensus.

### 2.8. Statistical Analysis

Statistical analyses were performed using GraphPad Prism version 10.0 (GraphPad Software, San Diego, CA, USA) and MedCalc Statistical Software version 22.0 (MedCalc Software Ltd., Ostend, Belgium).

Continuous variables were tested for normality using the Shapiro–Wilk test and expressed as mean ± standard deviation (SD) or median (interquartile range, IQR), as appropriate. Categorical variables were expressed as absolute values and percentages.

Between-group comparisons were conducted using the Student’s *t*-test or Mann–Whitney U test for continuous data, and the Chi-square or Fisher’s exact test for categorical data. Changes in cytokine levels over time (T_0_–T_2_) were analyzed using repeated-measures ANOVA with Bonferroni post hoc correction. Correlations between cytokine variation and clinical outcomes (pain, opioid use, LOS) were examined using Pearson’s correlation coefficient (r).

A two-tailed *p*-value < 0.05 was considered statistically significant. Post hoc power analysis confirmed that the study had >80% power to detect a 20% difference in IL-6 levels between groups at α = 0.05.

To minimize bias, all procedures were conducted by the same surgical and anesthetic teams using standardized protocols. Laboratory analyses were performed by blinded personnel. Although the study was observational, the prospective design, strict inclusion criteria, and standardized sampling times enhanced data reliability and internal validity.

Only patients with complete cytokine measurements at all predefined time points (T_0_, T_1_, and T_2_) were included in the final analysis.

Only patients with complete cytokine measurements at all predefined time points (T_0_, T_1_, and T_2_) were included in the final analysis. No imputation methods were applied for missing data.

## 3. Results

### 3.1. Baseline Characteristics

A total of 80 patients were included in the final analysis: 40 in the Lidocaine Group (LG) and 40 in the Control Group (CG). Baseline demographic and perioperative variables were comparable between groups, with no significant differences in age, BMI, ASA classification, anesthesia duration, or intraoperative blood loss (Table 1).

In addition to demographic and perioperative variables, baseline comorbidities, clinical history, and oncologic characteristics were comparable between groups and are summarized in Table 2.

Intraoperative analgesic management was standardized across both groups. Total intraoperative fentanyl consumption and the use of perioperative co-interventions are summarized in Table 3. Total intraoperative fentanyl consumption did not differ significantly between groups, and no additional opioid-sparing or anti-inflammatory adjuncts were used.

### 3.2. Perioperative Cytokine Dynamics

At baseline (T_0_), serum levels of IL-6, TNF-α, CRP and Fibrinogen were similar between groups (all *p* > 0.05). At 24 h postoperatively (T_2_), inflammatory markers increased significantly in both groups, with a markedly attenuated rise in patients receiving lidocaine (Table 4). Repeated-measures ANOVA confirmed significant time × treatment interactions for all cytokines (IL-6: *p* < 0.001; TNF-α: *p* = 0.001; CRP: *p* < 0.001).

**Figure 3:**
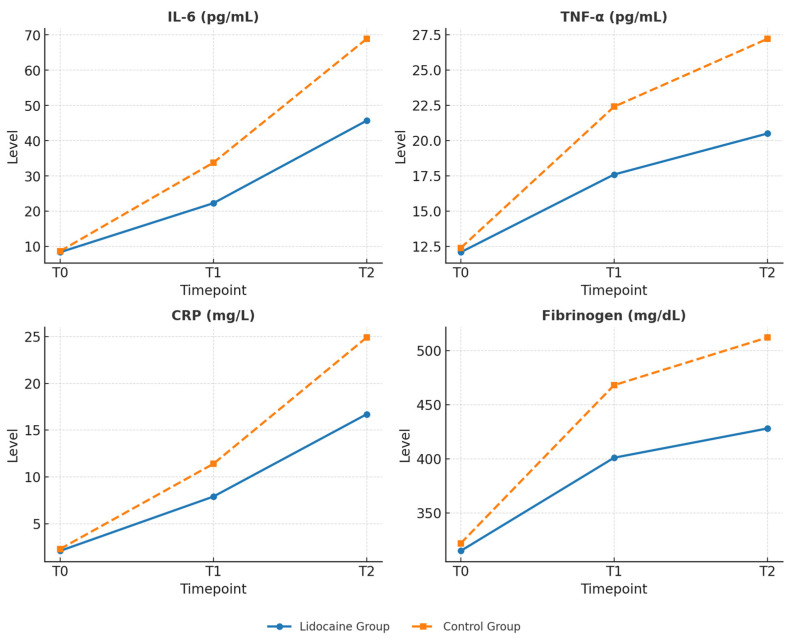
Perioperative evolution of IL-6, TNF-α, CRP, and fibrinogen in the two study groups. The solid blue line represents the Lidocaine group, while the dashed orange line represents the Control group. Data are presented as mean ± standard deviation (SD) at each time point (T0, T1, and T2).

The perioperative evolution of key inflammatory biomarkers is presented in Figure 3. The graphs illustrate changes in IL-6, TNF-α, CRP, and fibrinogen levels across all sampling points (T_0_, T_1_, and T_2_) in both study groups, showing a markedly attenuated postoperative rise in the Lidocaine Group compared to controls.

To further illustrate inter-individual variability in inflammatory responses, additional boxplots and individual patient trajectories for key cytokines are provided in the Supplementary Materials (Figures S1–S3).

### 3.3. Postoperative Pain and Opioid Consumption

Pain intensity (VAS) scores were significantly lower in the Lidocaine Group at all measured intervals (Table 5).

Total 24 h opioid consumption, expressed in morphine milligram equivalents (MME), was significantly reduced in the LG compared with the CG (8.9 ± 3.4 vs. 14.8 ± 5.2 mg, *p* < 0.001).

A moderate positive correlation was observed between IL-6 levels at 24 h and MME use (r = 0.47, *p* < 0.001).

### 3.4. Recovery Outcomes and Correlations

Patients who received intravenous lidocaine exhibited a noticeably faster postoperative recovery compared to controls. The return of gastrointestinal function occurred earlier, with a shorter time to oral intake (7.4 ± 2.6 h vs. 10.3 ± 3.8 h, *p* < 0.001) and an earlier passage of flatus (18.5 ± 6.2 h vs. 25.7 ± 8.9 h, *p* < 0.001). The overall hospital stay was also significantly reduced in the Lidocaine Group (2.9 ± 0.7 days vs. 3.6 ± 0.9 days, *p* = 0.003). Minor postoperative complications, including nausea, vomiting, and urinary retention, occurred less frequently among patients receiving lidocaine (10%) compared to those in the control group (25%), although this difference did not reach statistical significance (*p* = 0.08) (Table 6).

**Figure 4:**
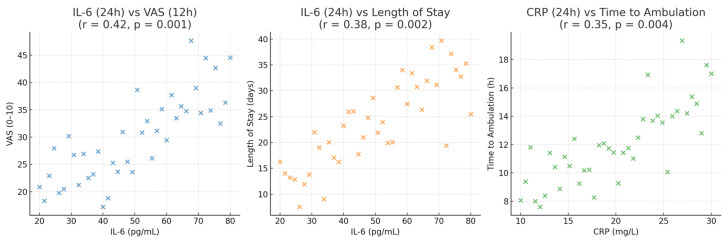
Associations between early postoperative inflammatory markers and clinical recovery parameters.

**Figure 5:**
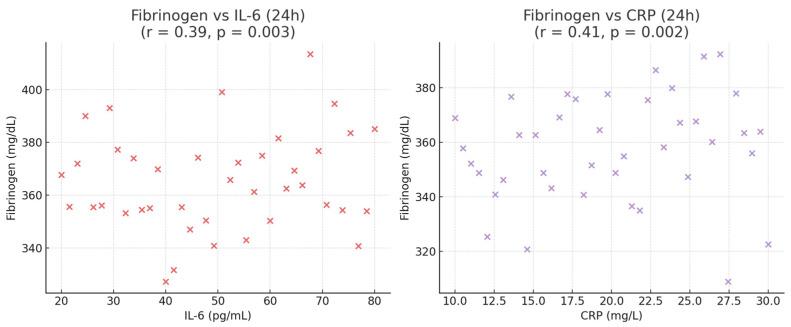
Correlation between fibrinogen levels and early postoperative inflammatory markers.

Correlation analyses further supported these findings. IL-6 levels at 24 h showed significant associations with pain intensity at 12 h (r = 0.42, *p* = 0.001) and with length of hospital stay (r = 0.38, *p* = 0.002). Similarly, CRP concentrations at 24 h correlated with time to ambulation (r = 0.35, *p* = 0.004) (Figure 4), while fibrinogen demonstrated moderate correlations with both IL-6 (r = 0.39, *p* = 0.003) and CRP (r = 0.41, *p* = 0.002), reflecting its role as part of the coordinated acute-phase response. (Figure 5). No significant correlation was observed between TNF-α levels and intraoperative parameters (*p* > 0.05). This association are all presented in Table 7.

Overall, these data indicate that lidocaine modulates the systemic inflammatory response in a concerted manner, acting on interconnected cytokine and acute-phase pathways rather than through isolated effects on individual biomarkers. Importantly, no lidocaine-related adverse events, such as arrhythmias, neurological symptoms, or hemodynamic instability, were observed during or after surgery, confirming the safety of the infusion protocol.

## 4. Discussion

The results of the study indicate that intravenous lidocaine, when administered during robotic-assisted radical prostatectomy, exerts a clear impact on the systemic inflammatory response to surgery. Patients receiving lidocaine exhibited significantly lower serum levels of IL-6, TNF-α, CRP and Fibrinogen within the first 24 h postoperatively, along with reduced pain intensity, lower opioid requirements, and faster recovery of bowel function and general mobility. These findings suggest that the perioperative effects of lidocaine extend beyond its traditional analgesic role, encompassing modulation of inflammatory and immune pathways activated by surgical stress.

Although robotic prostatectomy is a minimally invasive procedure, it is not biologically neutral [15]. Pneumoperitoneum, prolonged Trendelenburg positioning, and tissue manipulation inevitably trigger neuroendocrine and inflammatory activation [13]. Previous studies have demonstrated that even after laparoscopic or robotic procedures, circulating IL-6 [16,17,18] and CRP [17,18] levels rise significantly, confirming the systemic nature of the surgical stress response. The present results are consistent with this evidence but also demonstrate that lidocaine can partially blunt this response. The attenuation of cytokine release observed in patients receiving lidocaine indicates a potential contribution to restoring the balance between pro- and anti-inflammatory mechanisms during the early postoperative phase [4,5,8,19].

From a pathophysiological standpoint, these effects are likely driven by several complementary mechanisms. Beyond its local anesthetic action, lidocaine exerts systemic cellular effects, including inhibition of neutrophil activation, suppression of proinflammatory cytokine transcription, and modulation of the NF-κB signaling pathway [20]. Experimental data further suggest that lidocaine stabilizes cellular membranes and reduces oxidative stress by limiting mitochondrial dysfunction [5,21]. Together, these mechanisms contribute to a downregulation of the inflammatory cascade that typically follows surgical trauma. Clinically, this translates into measurable reductions in inflammatory markers and tangible benefits such as decreased pain intensity, reduced opioid need, and faster restoration of bowel activity [8].

The reduction in postoperative fibrinogen levels observed in the lidocaine group further supports the systemic anti-inflammatory potential of the drug [8,22]. As a key acute-phase reactant closely regulated by IL-6, fibrinogen not only reflects inflammatory activation but also contributes to perioperative coagulation and microcirculatory changes [23,24]. Its attenuation suggests that lidocaine may help maintain hemostatic balance and endothelial stability during the early recovery phase [3,25].

These observations are consistent with findings from other surgical specialties, particularly abdominal [26,27,28] and gynecologic procedures [11,29]. A previous study demonstrated that perioperative lidocaine infusions reduce IL-6 release and accelerate gastrointestinal recovery after open abdominal surgery [30]. Comparable benefits have been reported in laparoscopic colectomies, gynecologic oncology, and even cardiac surgery, where lidocaine administration was associated with decreased CRP levels and shorter hospital stays [31,32,33,34,35]. Evidence in robotic urologic surgery, however, remains limited [10,36]. The present study contributes to this growing field, indicating that lidocaine’s anti-inflammatory and recovery-promoting effects are equally relevant to minimally invasive prostatectomy, where the inflammatory burden is modest yet still clinically significant.

From a clinical perspective, the correlation between IL-6 concentrations and both opioid consumption and hospital stay duration is particularly noteworthy. IL-6 is a central mediator of the acute-phase response and is closely associated with heightened nociceptive sensitivity and delayed postoperative recovery [37]. Elevated IL-6 levels correlate with higher pain scores, prolonged ileus, and greater opioid requirements. By reducing IL-6 release, lidocaine appears to interrupt this cycle, promoting a biological environment conducive to improved healing and patient comfort. The parallel improvement in both biochemical and clinical parameters reinforces the rationale for incorporating lidocaine into multimodal anesthetic and analgesic strategies [4,30,38].

The immunologic dimension of lidocaine’s effects also warrants attention. Surgical trauma induces a transient immunologic imbalance characterized by systemic inflammation and temporary immune dysregulation [39,40,41]. Lidocaine has been shown in experimental and clinical settings to influence inflammatory signaling pathways. In the present study, the observed attenuation of proinflammatory biomarkers reflects short-term modulation of the postoperative inflammatory response. However, the study was not designed to assess immune cell function or oncologic outcomes, and any potential relevance in oncologic surgery should be interpreted cautiously and considered hypothesis-generating. Although long-term oncologic outcomes were not assessed, existing biological evidence suggests that perioperative inflammation may play a role in cancer-related processes; however, the present findings are limited to short-term biochemical and clinical outcomes and do not allow any inference regarding tumor behavior or oncologic prognosis [10,42,43]. Extending biomarker assessment beyond the first postoperative day would be particularly valuable to determine whether the observed early attenuation of inflammation translates into sustained immunologic modulation.

From an enhanced recovery after surgery (ERAS) perspective, these findings underscore the importance of inflammatory control in optimizing postoperative outcomes. Lidocaine infusion shortened the time to first flatus and oral intake, consistent with its established prokinetic effects on gastrointestinal function [44]. These effects may result from reduced sympathetic tone as well as decreased inflammatory interference with enteric neural signaling. The lower incidence of postoperative nausea and vomiting observed in the lidocaine group, though not statistically significant, aligns with the concept that stable hemodynamics, limited opioid exposure, and improved gut function [45] collectively enhance postoperative comfort and satisfaction [46].

In the context of multimodal perioperative management, several non-opioid agents have been investigated for their anti-inflammatory and opioid-sparing properties. Nonsteroidal anti-inflammatory drugs (NSAIDs) effectively reduce postoperative pain and inflammation but may be limited by gastrointestinal, renal, or bleeding risks, particularly in oncologic or elderly patients [47]. Corticosteroids, such as dexamethasone, exhibit potent anti-inflammatory effects and reduce postoperative nausea and pain; however, concerns regarding immunosuppression, hyperglycemia, and infection risk restrict their routine use [48].

Dexmedetomidine and low-dose ketamine have also been shown to attenuate perioperative inflammatory responses and reduce opioid consumption through central sympatholytic and NMDA receptor–mediated mechanisms, respectively [49]. Compared with these agents, intravenous lidocaine offers a unique combination of systemic anti-inflammatory, analgesic, and immunomodulatory effects, with a favorable safety profile when administered at subanesthetic doses. Its low cost, ease of administration, and limited impact on hemodynamics make lidocaine particularly attractive as an adjunct within enhanced recovery pathways.

In terms of anesthetic practice, intravenous lidocaine offers practical advantages. It is cost-effective, straightforward to administer, and, when dosed appropriately, highly safe. No adverse effects such as arrhythmias, hypotension, or neurological symptoms were observed in this cohort, confirming the safety of the infusion regimen employed. The total administered dose remained below toxic thresholds, and the infusion was discontinued at the end of surgery, consistent with ERAS recommendations. These features make lidocaine an attractive adjunct for multimodal anesthesia, particularly in settings prioritizing rapid recovery and cost efficiency [3,50].

The broader physiological implications of reducing surgical inflammation should also be considered. Beyond immediate postoperative comfort, systemic inflammation impacts endothelial integrity, coagulation, and microcirculatory function. Pharmacologic attenuation of these processes could potentially lower the risk of postoperative complications and improve tissue perfusion. Although biomarkers of endothelial activation or oxidative stress were not evaluated, the observed reductions in inflammatory cytokines may indirectly reflect enhanced vascular and immunologic stability throughout the perioperative period [51,52,53].

Within the context of existing literature, these results reinforce the growing evidence supporting lidocaine as an immunomodulatory adjunct in anesthesia. Meta-analyses and randomized controlled trials consistently demonstrate that lidocaine infusions reduce postoperative pain, opioid use, and inflammatory marker levels across diverse surgical settings. Most prior research has focused on open or laparoscopic abdominal procedures; demonstrating similar effects in robotic prostatectomy suggests that the benefits of lidocaine are independent of surgical approach, depending instead on its capacity to modulate the systemic neuroinflammatory response to tissue injury and anesthetic stress [8,54].

The integrative nature of lidocaine’s pharmacologic profile deserves particular emphasis. Its concurrent analgesic, anti-inflammatory, and immunomodulatory properties position it at the intersection of anesthesia, surgery, and perioperative medicine. In oncologic contexts, where perioperative inflammation and immune balance are increasingly recognized as relevant factors, agents that attenuate the acute inflammatory response may be of interest. In this study, lidocaine was associated with favorable short-term biochemical and clinical recovery parameters. However, its effects on long-term immune competence or cancer-related outcomes cannot be inferred from the present data [8].

The findings of this study highlight the potential for lidocaine infusion to become a standard component of multimodal analgesia in robotic prostatectomy. By blunting systemic inflammation and reducing opioid use, lidocaine may not only accelerate recovery but also contribute to an improved immunologic milieu, which could be particularly relevant in the oncologic context. Integrating lidocaine into Enhanced ERAS protocols may therefore offer both functional and biological benefits.

Strengths, limitations and future directions.

A major strength of this study lies in its prospective design and the homogeneity of the study population, which consisted exclusively of patients undergoing robotic-assisted radical prostatectomy performed by the same experienced surgical and anesthetic team. This uniformity minimized procedural variability and allowed a more accurate evaluation of lidocaine’s systemic effects. The integration of biochemical, clinical, and functional outcomes, including cytokine levels, pain scores, opioid use, and recovery metrics, provides a comprehensive view of how lidocaine modulates both the biological and clinical dimensions of postoperative recovery. Another notable strength is the focus on robotic urologic oncology, an area where evidence regarding systemic inflammatory modulation remains limited, despite the widespread adoption of robotic platforms.

However, several limitations should be acknowledged. First, the sample size, while adequate for detecting significant differences in cytokine levels and pain outcomes, may limit the statistical power for rarer events or subgroup analyses. Second, the study was conducted at a single center, which ensures protocol consistency but may affect external validity. In addition, the homogeneous study population, consisting exclusively of male patients undergoing robotic-assisted radical prostatectomy, further limits the generalizability of the findings. Although the observed anti-inflammatory and recovery-associated effects of intravenous lidocaine are consistent with data from other surgical specialties, caution is warranted when extrapolating these results to female patients, other oncologic procedures, or different surgical settings. The extent to which similar effects may be observed across diverse patient populations and types of surgery remains to be established. Although randomization was not performed, the two groups were comparable in demographic and perioperative variables, and strict inclusion criteria were applied to reduce selection bias. Another important limitation concerns the short observation window, restricted to the first 24 postoperative hours. While this time frame captures the early postoperative inflammatory response, it does not allow assessment of sustained or delayed immunologic effects. Longer follow-up periods, such as 72 h or even 7 days postoperatively, could provide a more comprehensive understanding of the temporal dynamics of inflammation and recovery. Future studies would benefit from longitudinal immune profiling, including anti-inflammatory cytokines (e.g., interleukin-10), lymphocyte subpopulation analysis, and later measurements of acute-phase reactants such as CRP and fibrinogen, in order to better characterize the immunomodulatory potential of intravenous lidocaine beyond the immediate postoperative period. In addition, given the non-randomized design, residual confounding by indication cannot be completely excluded, particularly with respect to intraoperative analgesic selection. Although baseline demographic and perioperative variables were similar between groups, unmeasured factors may have influenced group allocation. Finally, the study did not assess other potentially relevant biomarkers, such as interleukin-10, cortisol, or markers of oxidative stress, which could have further clarified the mechanistic pathways involved. Another limitation is the lack of a postoperative immune profiling beyond 24 h. Evaluating late immune markers or cytotoxic cell activity could better define the duration of lidocaine’s immunomodulatory effects. Although procedural consistency was ensured between groups, the observational non-randomized design precludes causal inference, and the use of standardized infusion practices without random allocation may have introduced residual performance bias. Although intraoperative analgesic management was standardized and comparable between groups, residual confounding related to individual opioid responsiveness cannot be entirely excluded.

The exclusion of patients with significant cardiac dysfunction (ejection fraction < 40%), moderate-to-severe renal impairment (eGFR < 45 mL/min/1.73 m^2^), or advanced liver disease (Child–Pugh class B/C) limits the generalizability of these findings to higher-risk surgical populations. Therefore, the results should be interpreted primarily in the context of patients with preserved organ function undergoing elective robotic-assisted radical prostatectomy.

Future research should aim to expand these findings in larger, multicenter randomized trials, including diverse patient populations and different types of surgical and oncologic procedures, in order to confirm the reproducibility and generalizability of the observed effects. Extending the observation period to include long-term oncologic outcomes, such as biochemical recurrence or immune competence, could help determine whether perioperative inflammation control influences cancer progression. In addition, mechanistic studies integrating transcriptomic or proteomic analyses might reveal new pathways through which lidocaine modulates immune signaling and tissue repair. Exploring its interaction with other elements of the ERAS protocol, as well as its potential synergy with regional anesthesia techniques, may further optimize perioperative care and patient recovery.

As an observational non-randomized study, this analysis is subject to potential selection bias and performance bias. Although standardized perioperative protocols were used and baseline characteristics were comparable between groups, residual confounding cannot be entirely excluded.

## 5. Conclusions

In summary, the present findings indicate that, in this prospective non-randomized observational study, intravenous lidocaine administered during robotic-assisted radical prostatectomy was associated with significant short-term anti-inflammatory effects and favorable postoperative recovery profiles. By attenuating perioperative increases in IL-6, TNF-α, CRP, and fibrinogen, lidocaine promotes a more balanced physiological response to surgical stress, resulting in reduced postoperative pain, lower opioid requirements, and faster recovery of gastrointestinal and overall function.

These findings, combined with its favorable safety profile, low cost, and ease of intraoperative administration, support the potential role of lidocaine as a valuable adjunct in multimodal perioperative care. Nevertheless, the absence of randomization limits causal interpretation, and the results should be viewed as associative rather than confirmatory. Beyond its well-established analgesic properties, lidocaine appears to contribute to the concept of “biological recovery”, a state in which optimal healing and immune equilibrium complement clinical recovery.

This integrative role highlights its potential to improve short-term postoperative outcomes, while any implications for long-term immune or oncologic prognosis remain speculative and require confirmation in future studies specifically designed to address these endpoints. Further large-scale, multicenter prospective studies are warranted to confirm and extend these observations.

## Figures and Tables

**Table 1:** Baseline demographic and perioperative characteristics of the study population.

Parameter	Lidocaine Group (*n* = 40)	Control Group (*n* = 40)	*p*-Value
Age (years) ^(a)^	64.2 ± 6.8	65.1 ± 7.1	0.58
BMI (kg/m^2^) ^(a)^	27.4 ± 2.9	27.9 ± 3.1	0.47
ASA physical status (I/II/III)	6/26/8	5/27/8	0.91
Duration of anesthesia (min) ^(a)^	195 ± 28	200 ± 31	0.42
Duration of surgery (min) ^(a)^	165 ± 26	170 ± 29	0.37
Estimated blood loss (mL) ^(a)^	220 ± 75	240 ± 80	0.29
Prostate volume (mL) ^(a)^	46.8 ± 11.4	48.2 ± 12.1	0.54

^(a)^ mean ± SD.

**Table 2:** Baseline comorbidities, clinical history, and oncologic characteristics of the study population.

Variable	Lidocaine Group (*n* = 40)	Control Group (*n* = 40)	*p*-Value
Hypertension	23 (57.5)	24 (60.0)	0.82
Diabetes mellitus type 2	9 (22.5)	10 (25.0)	0.79
Coronary artery disease/stable heart failure (EF > 40%)	6 (15.0)	7 (17.5)	0.76
History of cardiac arrhythmias,	4 (10.0)	5 (12.5)	0.72
Chronic kidney disease (stage 2–3a)	5 (12.5)	6 (15.0)	0.74
Chronic pulmonary disease (COPD/asthma/OSA)	7 (17.5)	8 (20.0)	0.77
Neurologic disease (stroke, Parkinson’s disease, epilepsy)	3 (7.5)	3 (7.5)	1.00
Current smokers	14 (35.0)	15 (37.5)	0.82
Former smokers	11 (27.5)	10 (25.0)	0.80
Prior major abdominal or pelvic surgery	8 (20.0)	9 (22.5)	0.79
Beta-blocker therapy	12 (30.0)	13 (32.5)	0.81
ACE inhibitor/ARB therapy	18 (45.0)	19 (47.5)	0.83
Statin therapy	16 (40.0)	17 (42.5)	0.82
Chronic NSAID use	5 (12.5)	6 (15.0)	0.74
Chronic corticosteroid therapy	0	0	—
Neoadjuvant oncologic therapy	0	0	—
Metastatic disease (M1)	0	0	—

Values are presented as number (percentage). No statistically significant differences were observed between groups.

**Table 3:** Intraoperative analgesic management.

Parameter	Lidocaine (*n* = 40)	Control (*n* = 40)	*p*
Total intraoperative fentanyl (µg)	185 ± 42	192 ± 45	0.46
Additional opioids	0	0	—
Ketamine	0	0	—
NSAIDs/acetaminophen	0	0	—
Gabapentinoids	0	0	—
TAP block/local infiltration	0	0	—

**Table 4:** Evolution of inflammatory biomarkers over time.

Biomarker	Timepoint	Lidocaine Group	Control Group	*p*-Value
IL-6 ^(a)^	T_0_	8.4 ± 2.1	8.7 ± 2.4	0.61
	T_1_	22.3 ± 7.6	33.8 ± 8.9	<0.01 *
	T_2_	45.7 ± 10.8	68.9 ± 12.6	<0.01 *
TNF-α ^(a)^	T_0_	12.1 ± 3.3	12.4 ± 3.0	0.72
	T_1_	17.6 ± 4.2	22.4 ± 5.1	<0.01 *
	T_2_	20.5 ± 5.1	27.2 ± 6.4	<0.01 *
CRP ^(b)^	T_0_	2.1 ± 0.6	2.3 ± 0.7	0.31
	T_1_	7.9 ± 3.2	11.4 ± 4.1	<0.01 *
	T_2_	16.7 ± 5.3	24.9 ± 7.1	<0.01 *
Fibrinogen ^(c)^	T_0_	315 ± 48	322 ± 51	0.44
	T_1_	401 ± 66	468 ± 72	<0.01 *
	T_2_	428 ± 58	512 ± 75	<0.01 *

^(a)^ (pg/mL) ^(b)^ (mg/L); ^(c)^ mg/dL; T_0_—baseline; T_1_—end of surgery; T_2_—24 h postoperative; The values are reported as mean ± SD; * Significant *p*-value.

**Table 5:** Postoperative pain scores and opioid consumption.

Parameter	Lidocaine Group (*n* = 40)	Control Group (*n* = 40)	*p*-Value
VAS 4 h	3.1 ± 1.0	4.6 ± 1.2	<0.001 *
VAS 12 h	2.5 ± 0.9	3.9 ± 1.1	<0.001 *
VAS 24 h	1.8 ± 0.7	2.7 ± 0.8	0.002 *
Total opioid use (MME)	8.9 ± 3.4	14.8 ± 5.2	<0.001 *

The values are reported as mean ± SD; * Significant *p*-value; MME = Morphine Milligram Equivalent.

**Table 6:** Postoperative recovery parameters in patients receiving intravenous lidocaine versus controls.

Parameter	Lidocaine Group (*n* = 40)	Control Group (*n* = 40)	*p*-Value
Time to oral intake (hours) ^(a)^	7.4 ± 2.6	10.3 ± 3.8	<0.001 *
Time to first flatus (hours) ^(a)^	18.5 ± 6.2	25.7 ± 8.9	<0.001 *
Length of hospital stay (days) ^(a)^	2.9 ± 0.7	3.6 ± 0.9	0.003 *
Minor postoperative complications (%)	10%	25%	0.08

^(a)^ mean ± SD; * Significant *p*-value.

**Table 7:** Correlation analysis between inflammatory biomarkers and clinical recovery parameters.

Biomarker (24 h)	Clinical/Laboratory Parameter	Correlation Coefficient (r)	*p*-Value	Interpretation
IL-6	VAS pain score at 12 h	0.42	0.001 *	Moderate positive correlation
IL-6	Length of hospital stay	0.38	0.002 *	Moderate positive correlation
CRP	Time to ambulation	0.35	0.004 *	Moderate positive correlation
Fibrinogen	IL-6	0.39	0.003 *	Moderate positive correlation
Fibrinogen	CRP	0.41	0.002 *	Moderate positive correlation
TNF-α	Intraoperative variables	—	>0.05 *	No significant correlation

* Significant *p*-value, Abbreviation: IL-6, interleukin-6; CRP, C-reactive protein; TNF-α, tumor necrosis factor-alpha; VAS, Visual Analogue Scale.

## Data Availability

Data is contained within the article or Supplementary Material.

## Supplementary Materials

The following supporting information can be downloaded at: https://www.mdpi.com/article/10.3390/medicina62010068/s1. Figure S1: Individual perioperative trajectories of serum IL-6 levels in patients undergoing robotic-assisted radical prostatectomy. (A) Lidocaine Group; (B) Control Group. Each line represents an individual patient. A consistently attenuated postoperative increase is observed in the Lidocaine Group compared with controls; Figure S2: Boxplots illustrating inter-individual variability of serum IL-6 concentrations at baseline (T_0_), end of surgery (T_1_), and 24 hours postoperatively (T_2_) in the Lidocaine and Control groups. Boxes represent interquartile range with median; whiskers indicate minimum and maximum values; Figure S3: Individual perioperative trajectories of serum TNF-α levels in patients undergoing robotic-assisted radical prostatectomy. (A) Lidocaine Group; (B) Control Group. Each line represents an individual patient; Figure S4: Boxplots illustrating inter-individual variability of serum TNF-α concentrations at baseline (T_0_), end of surgery (T_1_), and 24 hours postoperatively (T_2_) in the Lidocaine and Control groups. Boxes represent interquartile range with median; whiskers indicate minimum and maximum values.
