# Supplementary material for: Systemic Anti-Inflammatory and Immunomodulatory Effects of Intravenous Lidocaine During Robotic-Assisted Radical Prostatectomy: A Prospective Observational Study
Source: Medicina (Kaunas). 2025 Dec 28;62(1):68. doi: 10.3390/medicina62010068 (PMC12842614; doi:10.3390/medicina62010068)
Supplement: Supplementary file 1 [file medicina-62-00068-s001.zip › medicina-4055272-supplementary.pdf]

**Supplementary Figure S1.** Individual perioperative trajectories of serum IL-6 levels in patients undergoing robotic-assisted radical prostatectomy. (A) Lidocaine Group; (B) Control Group. Each line represents an individual patient. A consistently attenuated postoperative increase is observed in the Lidocaine Group compared with controls.

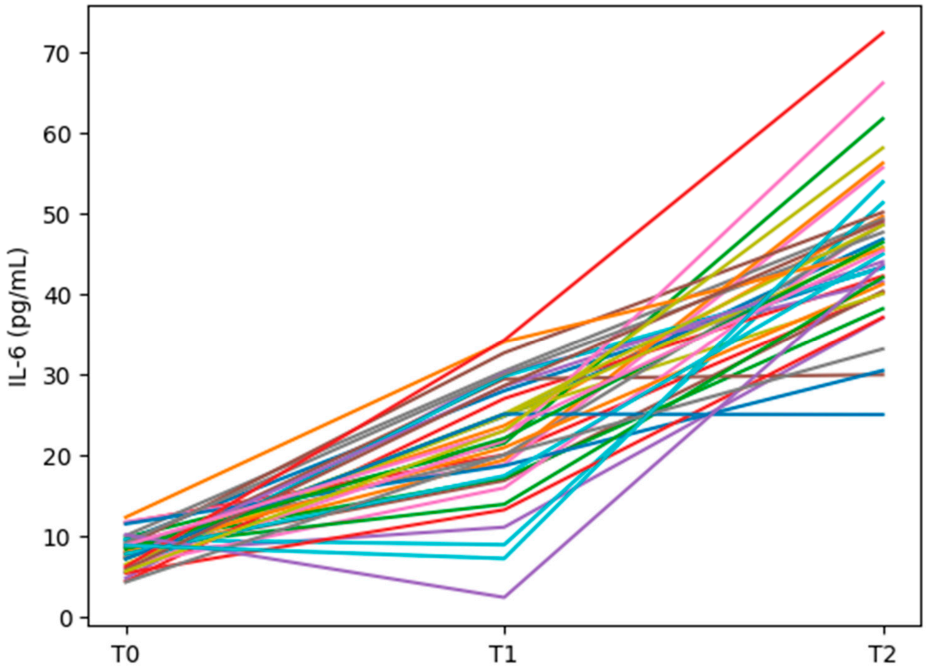


**Supplementary Figure S1A**

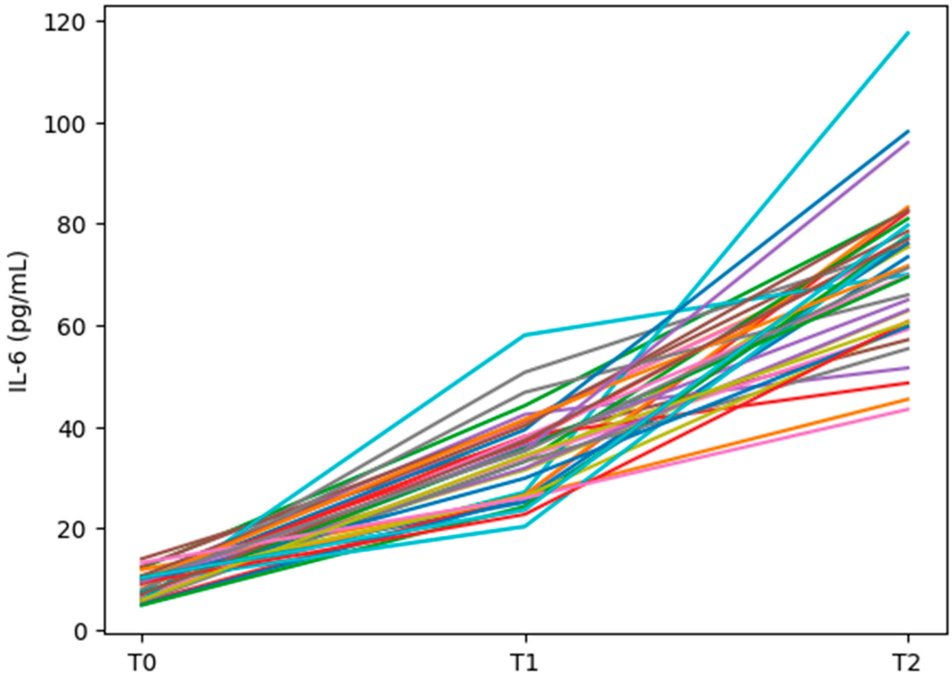


**Supplementary Figure S1B**

Supplementary Figure S2. Boxplots illustrating inter-individual variability of serum IL-6 concentrations at baseline ($T_0$), end of surgery ($T_1$), and 24 hours postoperatively ($T_2$) in the Lidocaine and Control groups. Boxes represent interquartile range with median; whiskers indicate minimum and maximum values.

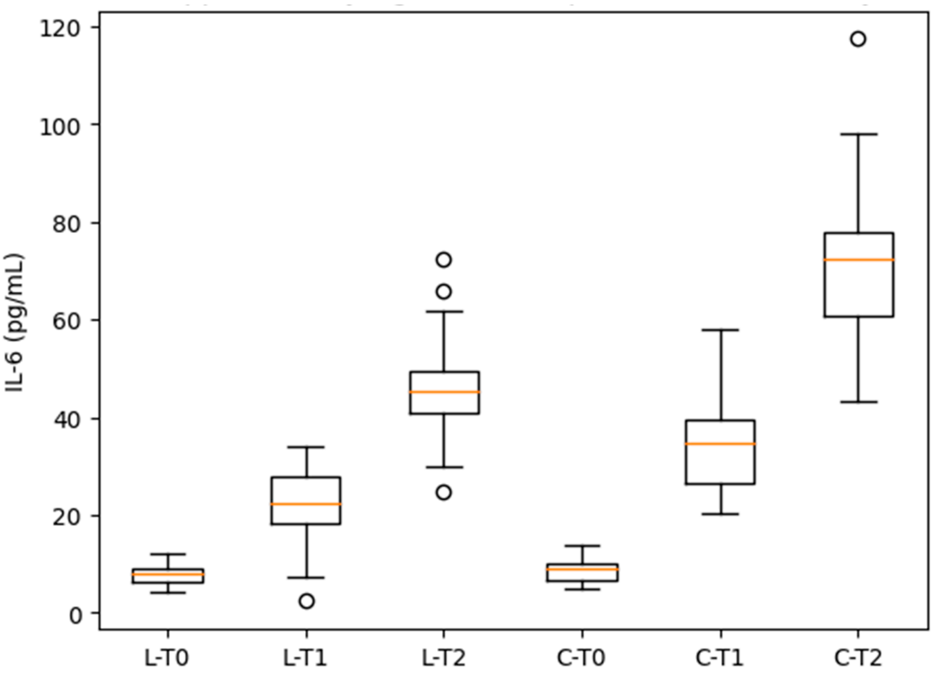


**Supplementary Figure S2**

**Supplementary Figure S3.** Individual perioperative trajectories of serum TNF-$\alpha$ levels in patients undergoing robotic-assisted radical prostatectomy. (A) Lidocaine Group; (B) Control Group. Each line represents an individual patient.

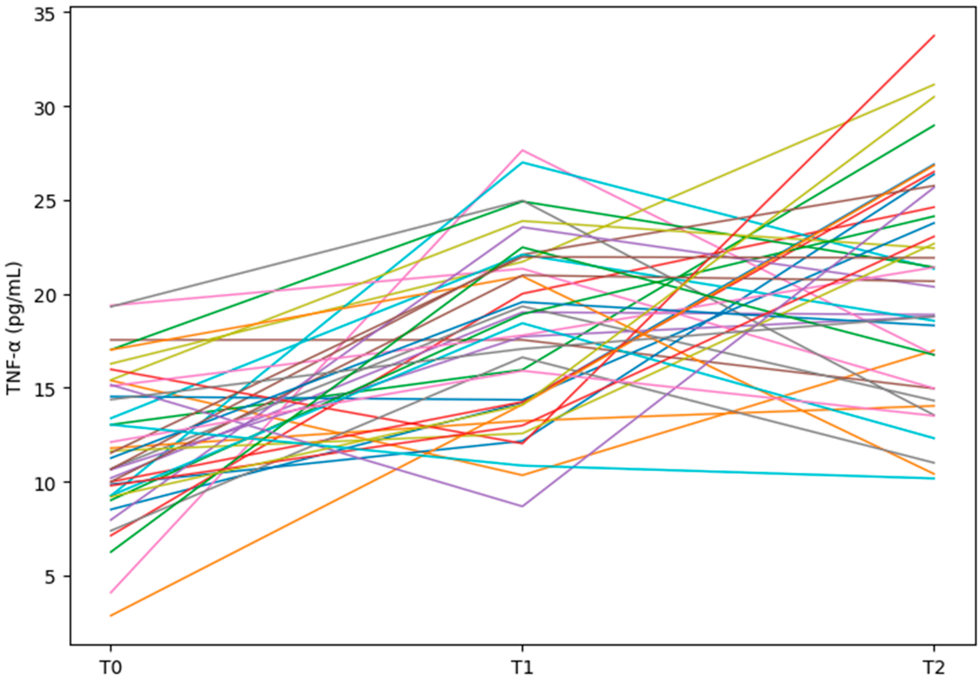


**Supplementary Figure S3A**

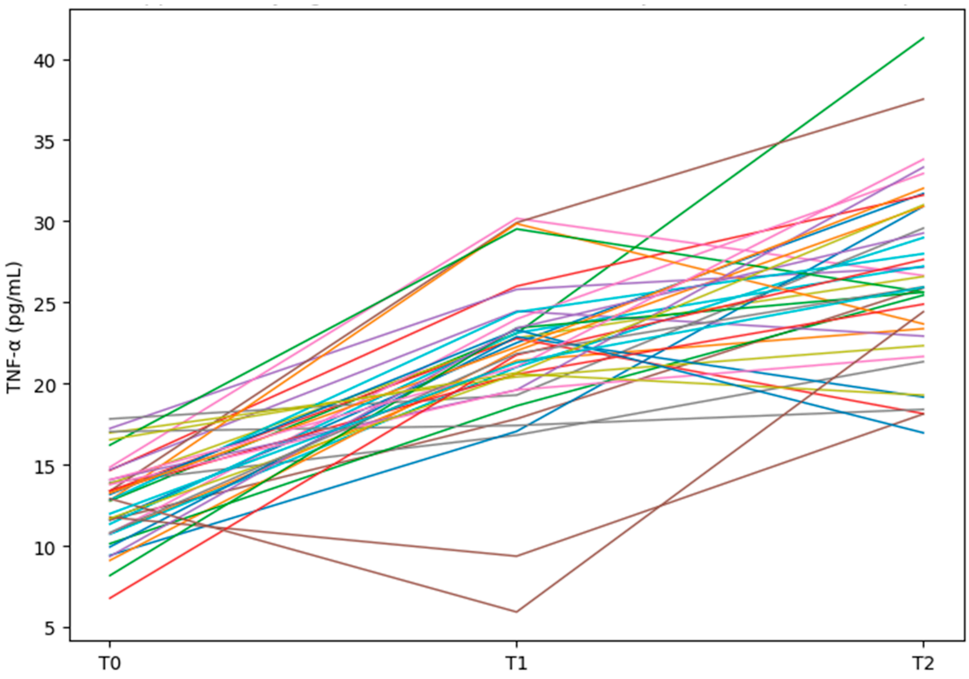


**Supplementary Figure S3B**

**Supplementary Figure S4.** Boxplots illustrating inter-individual variability of serum TNF-$\alpha$ concentrations at baseline ($T_0$), end of surgery ($T_1$), and 24 hours postoperatively ($T_2$) in the Lidocaine and Control groups. Boxes represent interquartile range with median; whiskers indicate minimum and maximum values.

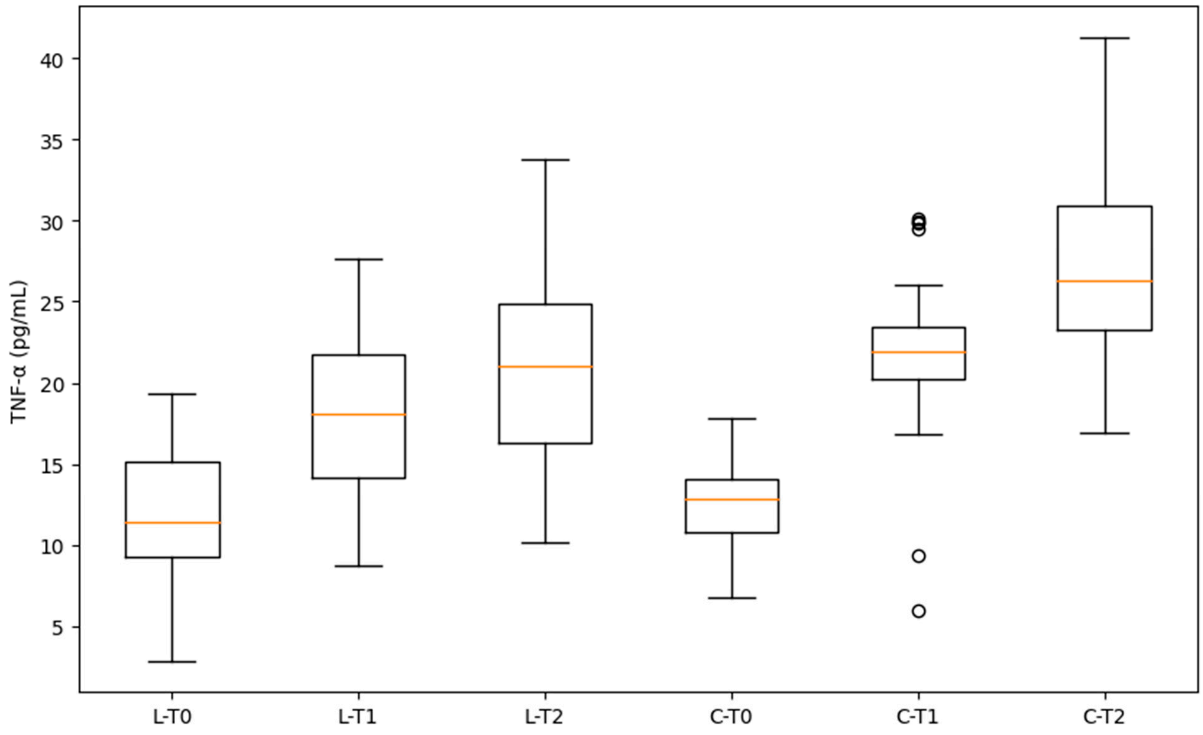


**Supplementary Figure S4**
